# Bilateral Muscle Architecture Differences in a Rabbit Model of Unilateral Hindlimb Transtibial Amputation: A Pilot Study

**DOI:** 10.3390/muscles5030065

**Published:** 2026-09-16

**Authors:** Caleb Stubbs, Patrick T. Hall, Allison J. Nelson, Samantha Z. Bratcher, Alicia A. Matavosian, Cheryl B. Greenacre, Bryce J. Burton, Stacy M. Stephenson, Remigiusz M. Grzeskowiak, Alisha P. Pedersen, Xiaojuan Zhu, David E. Anderson, Dustin L. Crouch

**Affiliations:** 1 Department of Biomedical Engineering, Tickle College of Engineering, University of Tennessee, Knoxville, TN 37996, USA; 2 College of Veterinary Medicine, University of Tennessee, Knoxville, TN 37996, USAdander48@utk.edu (D.E.A.); 3 Department of Surgery, Division of Plastic and Reconstructive Surgery, University of Tennessee Graduate School of Medicine, Knoxville, TN 37920, USA; 4 Office of Innovative Technologies, University of Tennessee, Knoxville, TN 37996, USA

**Keywords:** residual limb, muscle architecture, muscle plasticity, amputation, rabbit model

## Abstract

Residual muscles after amputation experience mechanical unloading, loss of distal tendon insertion, and reduced excursion, conditions known to cause muscle degeneration. However, the architecture of residual muscles following amputation remains largely unreported. In this pilot, exploratory study, five healthy, skeletally mature, male New Zealand white rabbits underwent unilateral hind-paw ankle disarticulation. The contralateral intact limb served as the within-subject control. Six hindlimb muscles that cross the intact biological ankle were harvested bilaterally at four weeks post-amputation, and muscle mass, length, fiber length, pennation angle, sarcomere length, optimal fiber length (OFL), and physiologic cross-sectional area (PCSA) were measured. All statistical comparisons were based on raw, paired differences in values between sides. Muscle mass and OFL were, on average, lower on the residual side for all muscles; raw differences between sides were significant for mass of LG (*p* = 0.029) and FDS (*p* = 0.026) and for OFL of LG (*p* = 0.026). Based on percent difference between sides, degeneration was most severe in the soleus, which had 63 ± 26% less mass and 51 ± 40% lower optimal fiber length in the residual limb than in the intact contralateral limb. Despite lower mass, average PCSA was generally preserved or increased (though differences were not statistically significant) across all muscles except FDS; this was possibly attributable to disproportionately greater reductions in optimal fiber length than in mass. These results have important potential implications for prosthesis function and, therefore, motivate additional research to identify influential factors and evaluate clinical strategies for preserving residual muscle architecture.

## 1. Introduction

Acquired (i.e., traumatic or surgical) amputation severely disrupts many organ systems. For example, muscles and tendons are severed and released from their distal insertion points so that they no longer cross the missing joints. Despite the disruption, muscles in the residual limb typically retain some useful, coordinated contractile function [1] that can be leveraged in several ways. For example, electromyograms, the sum of neuromotor action potentials that initiate residual muscle contraction, have been used for decades to control myoelectric prosthetic limbs [2,3]. The coordinated contraction and mechanical interaction of surgically connected agonist-antagonist residual muscle pairs has been shown to restore proprioception of missing joints [4]. Physical attachment of residual muscles to external prostheses [5,6] and, more recently, internal prostheses [7], transmits residual muscle contractile forces and displacements directly to a prosthesis. Finally, multi-articular residual muscles may still cross and contribute to movement at one or more residual biological joints.

Because residual muscles can support prosthetic and residual limb function in many ways, knowing how the structure and function of the residual muscles change following amputation is critically important for preserving or enhancing patient functional ability, independence, and quality of life. The volume and composition of residual muscles have been reported in several studies. In lower-limb amputees, muscle volumes in the residual and contralateral intact limbs either increased (hypertrophy) or decreased (atrophy), reflecting either greater (compensatory) or lesser use, respectively, depending on amputation level [8]. Volumes of muscles even farther removed from the amputation site, such as trunk muscles, may be affected due to altered or compensatory movement strategies [9]. Residual muscles in patients with transtibial amputation had a greater percentage of intramuscular fat, consistent with muscle atrophy, than in intact limbs and controls [10]. Reports of muscle changes in human patients can be confounded by intra-patient variability of factors such as time since amputation, amputation level, physical activity level and other patient-specific factors such as age, sex, and anthropometry, though studies attempt to account for some of these factors [8].

Except for muscle volume, changes in muscle architecture properties following amputation, besides volume, remain unknown. Muscle architecture properties, such as mass, fiber length, optimal fiber length, and physiologic cross-sectional area, are correlated with the functional attributes of muscle such as excursion (e.g., length change), shortening velocity, and contractile force [11]. Following amputation, it is expected that residual muscles retract and essentially become immobilized in a shortened position. Residual muscles that cross an intact residual joint will still experience forces and length changes, though they are likely altered from those of the intact limb. Such changes in geometry and use are similar to those of tenotomy [4] and limb immobilization [5], both of which lead to atrophy, reduced normalized fiber length (i.e., fewer sarcomeres in series), and reduced fiber cross-sectional area. Knowing how muscle architecture changes following amputation could (1) inform the clinical management and rehabilitation of people with amputation, (2) improve patient function with prostheses, and (3) advance emergent methods for directly interfacing muscles with prostheses.

The objective of our pilot, exploratory study was to quantify muscle architecture bilaterally in select residual muscles (gastrocnemius, soleus, tibialis cranialis, extensor digitorum, and flexor digitorum superficialis) four weeks after unilateral hind-paw ankle disarticulation; the contralateral intact limb served as a control. We hypothesized that muscle mass and physiologic cross-sectional area would be lower in the residual limb than in the contralateral intact limb. The study was part of a larger project to develop and test an implantable foot-ankle prosthesis [7,12] to which the residual tibialis cranialis and triceps surae muscles are attached. Therefore, a secondary, exploratory aim was to determine whether suturing the tendons of these muscles to the distal end of the tibia, thereby approximately preserving their pre-amputation in situ lengths (though muscle length was not measured intraoperatively), could potentially preserve the muscles’ architecture [13].

## 2. Methods

All procedures were approved by the University of Tennessee Institutional Animal Care and Use Committee (Protocol #2594). Five healthy, skeletally mature, 13-week-old male New Zealand white (NZW) rabbits (mean body mass: 3.2 ± 0.3 kg; supplier: Charles River, Wilmington, MA, USA) were individually housed in standard rabbit enclosures with ad libitum access to food and water. The sample size was chosen to provide preliminary estimates of effect size and variance for planning a future adequately powered study. Animals were acclimated to the facility for at least one week prior to surgery. All rabbits underwent unilateral (right) hind paw disarticulation of the tibiotarsal (ankle) joint; the contralateral intact limb served as the within-subject control. The operated side was standardized to ensure the surgeon’s familiarity with the anatomy and minimize a potential source of variation in this small pilot study. Each animal was considered one experimental unit.

General anesthesia was induced with an intramuscular injection of midazolam (1 mg/kg) and hydromorphone (0.1 mg/kg), and maintained via isoflurane inhalation (1–3%) through a nose cone for the duration of surgery. Depth of anesthesia was initially confirmed by pedal withdrawal reflex, then monitored continuously via heart rate, respiratory rate, and body temperature. The surgical site was shaved, cleaned with chlorohexidine and isopropyl alcohol, and draped with sterile surgical drapes. The skin surrounding the ankle and extending approximately 2–3 cm distally was retracted to expose the underlying tendons and joint.

At the level of the ankle, a hemostat was clamped to the insertion tendons of the gastrocnemius and soleus (i.e., the Achilles tendon) and the tibialis cranialis muscles to limit their proximal retraction. The insertion tendons were cut distal to the hemostat or, for unclamped muscles, at the level of the ankle. The remaining tissues connecting the tibia and fibula to the foot were cut and the hind paw was removed. The insertion tendons of the tibialis cranialis and triceps surae muscles were sutured to the distal end of the tibia; the muscles were sutured so that their lengths approximated their pre-amputation lengths, though the lengths of the muscles were not measured after suturing. The retracted skin was replaced and sutured together over the end of the shank.

Immediately after surgery, the limb was bandaged up to the base of the knee (without immobilizing the knee) for about 2 weeks until the incision healed. The bandage was changed at least once every three days. First, silver sulfadiazine (SSD) topical cream was applied over the incision to prevent infection. The limb was then bandaged using, from inner to outer layers, non-adherent dressing (Telfa, Covidien, Mansfield, MA, USA), undercast padding, elastic bandage wrap, elastic tape (ELASTIKON, Johnson & Johnson, New Brunswick, NJ, USA), and bandage tape to protect the incision site. We administered an analgesic of either buprenorphine (0.03 mg/kg) subcutaneously or hydromorphone (0.2 mg/kg) intramuscularly every 6 h for at least 72 h post-surgery; antibiotics (enrofloxacin 5 mg/kg diluted) either subcutaneously or orally every 12 h for at least 7 days post-surgery; and an anti-inflammatory drug (meloxicam 0.6 mg/kg) either subcutaneously or orally every 24 h for at least 7 days post-surgery.

All rabbits were euthanized 4 weeks after amputation by anesthetic barbiturate overdose. The residual and contralateral intact hindlimbs were detached at the hip and placed in a custom jig that held the knee joint at 90° flexion to standardize the fixation position across animals. Hindlimbs were fixed in 10% neutral buffered formalin for a minimum of 3 days, then transferred to 70% ethanol for a minimum of 3 days prior to dissection.

No formal strategy (e.g., randomization, blinding) was used to minimize potential confounders. The lateral gastrocnemius (LG), medial gastrocnemius (MG), soleus (SO), tibialis cranialis (TA), extensor digitorum (ED), and flexor digitorum superficialis (FDS) muscle-tendon units were dissected from the bone. The tendons were removed from each muscle at the muscle–tendon junction using sharp dissection. After blotting muscles dry with a paper towel, we measured muscle length using a digital caliper (resolution 0.01 mm) and mass using a digital scale (resolution 0.001 g). The muscle mid-belly was cut parallel to the fibers, and muscle fiber length ($l_{f}$) was measured using a digital caliper. We measured muscle fiber length along the direction of the muscle fibers at the mid-belly of each muscle. Sarcomere lengths were measured by laser diffraction [14] using a He-Ne laser (HNLS008L, Thorlabs, Newton, NJ, USA). Fiber bundles were teased from the cut mid-belly surface and transilluminated with the laser beam; the resulting diffraction pattern was projected onto a screen at a known distance. Measured sarcomere length ($l_{s}$) was calculated as:

(1) $$l_{s}=\frac{n\lambda }{\left(sin(tan^{-1}(x_{n}/h))\right)}$$

where $x_{n}$ is the measured radius of the band (mm), n is the order of the band measured, h is the distance between the muscle fiber and the screen, and λ is the wavelength of the laser (λ = 632.8 nm). For each muscle, eight to ten sarcomere measurements were recorded and averaged.

Muscle fiber pennation angle (θ) was measured from digital photographs, taken perpendicular to the plane of the muscle fibers, using image processing software (ImageJ version 1.53, NIH). Optimal fiber length ($l_{f}^{0}$), also called normalized fiber length, was calculated as:

(2) $$l_{f}^{0}=l_{f}*\left(\frac{l_{s}^{0}}{l_{s}}\right)$$

where $l_{s}^{0}$ is the optimal sarcomere length (2.2 μm, from [15]) and $l_{s}$ is the measured sarcomere length. The physiologic cross-sectional area (PCSA) was calculated from the measured muscle mass (m) and fiber length using the following formula:

(3) $$PCSA=\frac{m\;*\;cos\;(\theta )}{(l_{f}^{0})\;*\;\rho }$$

where ρ is the density of skeletal muscle; we used the value ρ= 1.054 g/cm^3^, reported previously [15]. Optimal fiber length is proportional to the range of muscle lengths over which the muscle can produce active force [11,16], and PCSA is proportional to the maximum force a muscle can produce [17].

Data from all animals (n = 5) were included in the statistical analysis. All analyses were based on the raw difference (∆) in measured values between sides:

(4) $$∆=x_{residual}-x_{intact}\;$$

The normality of ∆ values was assessed for all data grouped across animals, muscles, and parameters using the Shapiro–Wilk test and QQ plots. Because the differences were normally distributed, a one-sample Student’s *t*-test was used to determine whether the difference for each muscle differed significantly from zero. The resulting *p*-values were adjusted for multiple comparisons using the false discovery rate (FDR) method (SAS Proc Multtest, version 9.4). The raw differences were compared among muscles using a mixed-effects ANOVA with animal as a random blocking factor (SAS Proc Glimmix). Least-squares means were computed and post hoc multiple pairwise comparisons for the ANOVA model were performed using the Tukey HSD method. The Shapiro–Wilk W and QQ normality plots were used to evaluate the normality of ANOVA residuals. A Levene’s test was used to assess the equality of variances for the residuals. All statistical assumptions regarding normality and equality of variances were met. Statistical significance was set at *p* < 0.05. All analyses were performed using SAS version 9.4, release TS1M8 (SAS Institute Inc., Cary, NC, USA).

To assess the magnitude of bilateral differences relative to values measured from the intact limb, we computed the within-subject percent difference (%∆) in each architectural property between the residual and intact limbs as:

(5) $$\%∆=\left(\frac{x_{residual}-x_{intact}}{x_{intact}}\right)\times 100$$

For all muscles, muscle blocks from the muscle mid-belly were paraffin-embedded, sectioned perpendicular to the long axis of the fibers, and stained with hematoxylin and eosin (H&E). Sections were imaged at 10× magnification using a microscope (BZ-X810, Keyence, Itasca, IL, USA). Muscle histology images for all muscles were assessed qualitatively by a single observer for muscle fiber diameter and shape.

## 3. Results

**Figure 1:**
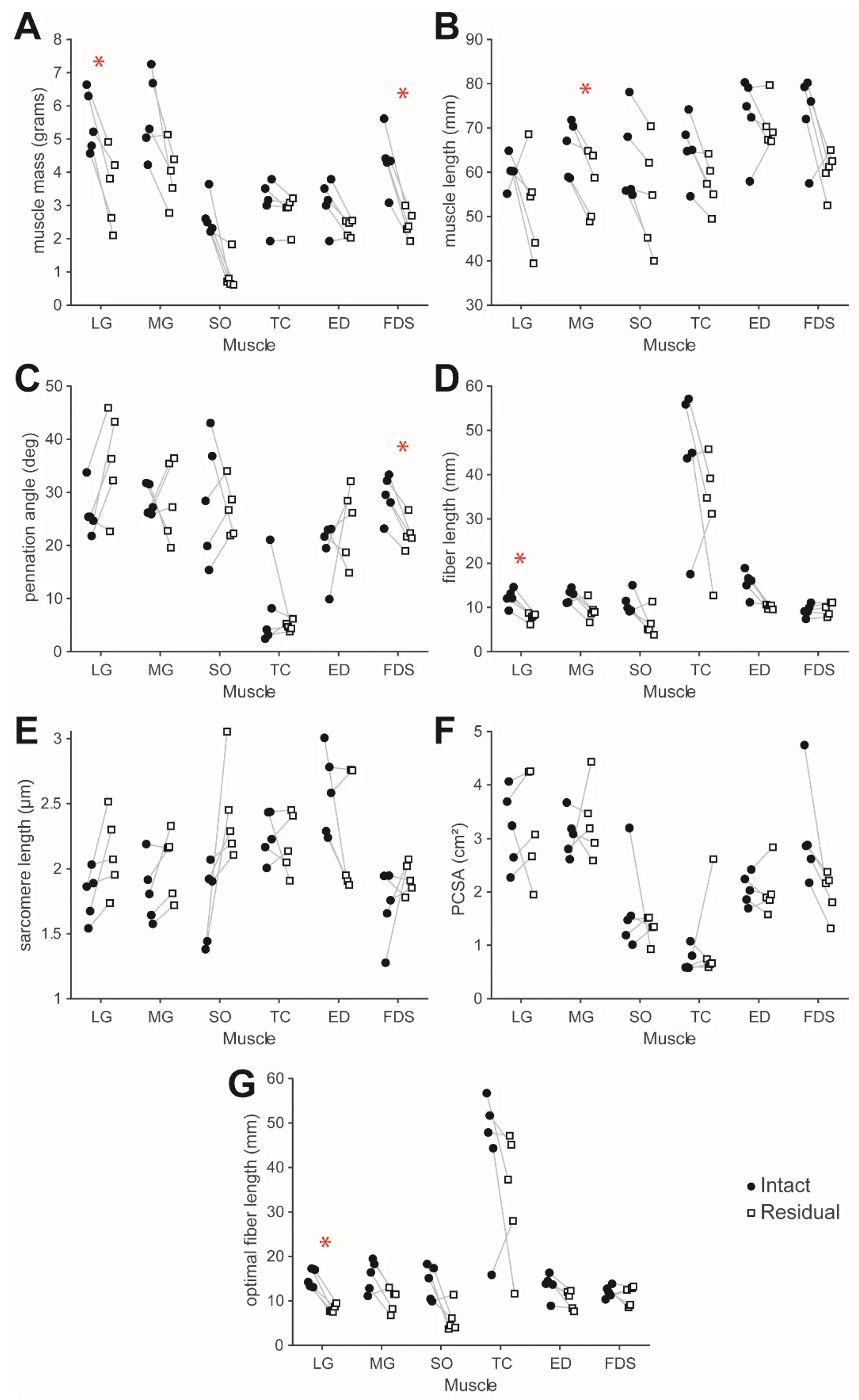
Muscle architecture variables, including (**A**) muscle mass, (**B**) muscle length, (**C**) pennation angle, (**D**) fiber length, (**E**) sarcomere length, (**F**) physiologic cross-sectional area (PCSA), and (**G**) optimal fiber length. * Raw difference between residual and contralateral intact limbs (Residual-Intact) was significantly different from zero (*p* < 0.05) based on a 1-sample *t*-test with FDR-adjusted *p*-value.

On average, mass was lower in the residual limb than in the contralateral intact limb for all muscles. However, bilateral difference in mass was statistically significant for LG (Δ = −1.97 ± 0.67 g, *p* = 0.029, 95% CL [−2.8, −1.14] g) and FDS (Δ = −1.89 ± 0.54 g, *p* = 0.026, 95% CL [−2.57, −1.22] g) (Table 1, Figure 1A).

As with mass, muscle length, fiber length, and optimal fiber length were also generally lower, on average, in the residual limb than in the contralateral intact limb for all muscles. Muscle length was significantly different between sides only for MG (Δ = −8.10 ± 3.55 mm, *p* = 0.049, 95% CL [−12.51, −3.69] mm) (Table 1, Figure 1B). For LG, fiber length (Δ = −4.36 ± 1.33 mm, *p* = 0.026, 95% CL [−6, −2.71] mm) and optimal fiber length (Δ = −6.74 ± 2.05 mm, *p* = 0.026, 95% CL [−9.28, −4.19] mm) were significantly different between sides (Table 1, Figure 1D,G).

Pennation angle was significantly lower between sides only for FDS (Δ = −7.06 ± 2.60 degrees, *p* = 0.031, 95% CL [−10.29, −3.83] degrees) (Table 1, Figure 1C).

Relative to the intact limb, the SO muscle exhibited the largest bilateral percent differences in average mass (%Δ = −63 ± 26%) and optimal fiber length (−51 ± 40%), though raw differences for these variables were not statistically significant (*p* = 0.072 and *p* = 0.129, respectively). Corresponding to significant raw differences in mass and optimal fiber length, LG also exhibited large percent differences in these variables (−37 ± 14% for mass, −44 ± 8% for optimal fiber length).

Despite lower average muscle mass on the residual side for all muscles, average bilateral differences in PCSA varied among muscles, though raw bilateral differences in PCSA were not statistically significant (Table 1, Figure 1F). Specifically, mean PCSA was about the same or higher on the residual side for LG (3 ± 24%), MG (11 ± 35%), SO (−4 ± 42%), TC (45 ± 104%), and ED (−2 ± 15%), but lower on the residual side for FDS (−33 ± 15%).

The mixed-effects ANOVA indicated significance for parameters mass (F = 3.88, *p* = 0.010) and sarcomere length (F = 3.51, *p* = 0.016). The post-hoc test revealed that bilateral mass differences were significantly different between the TC and FDS muscles (t = 3.21, *p* = 0.039). For sarcomere length, bilateral differences were significantly different between the SO and ED muscles (t = 3.9, *p* = 0.008).

**Figure 2:**
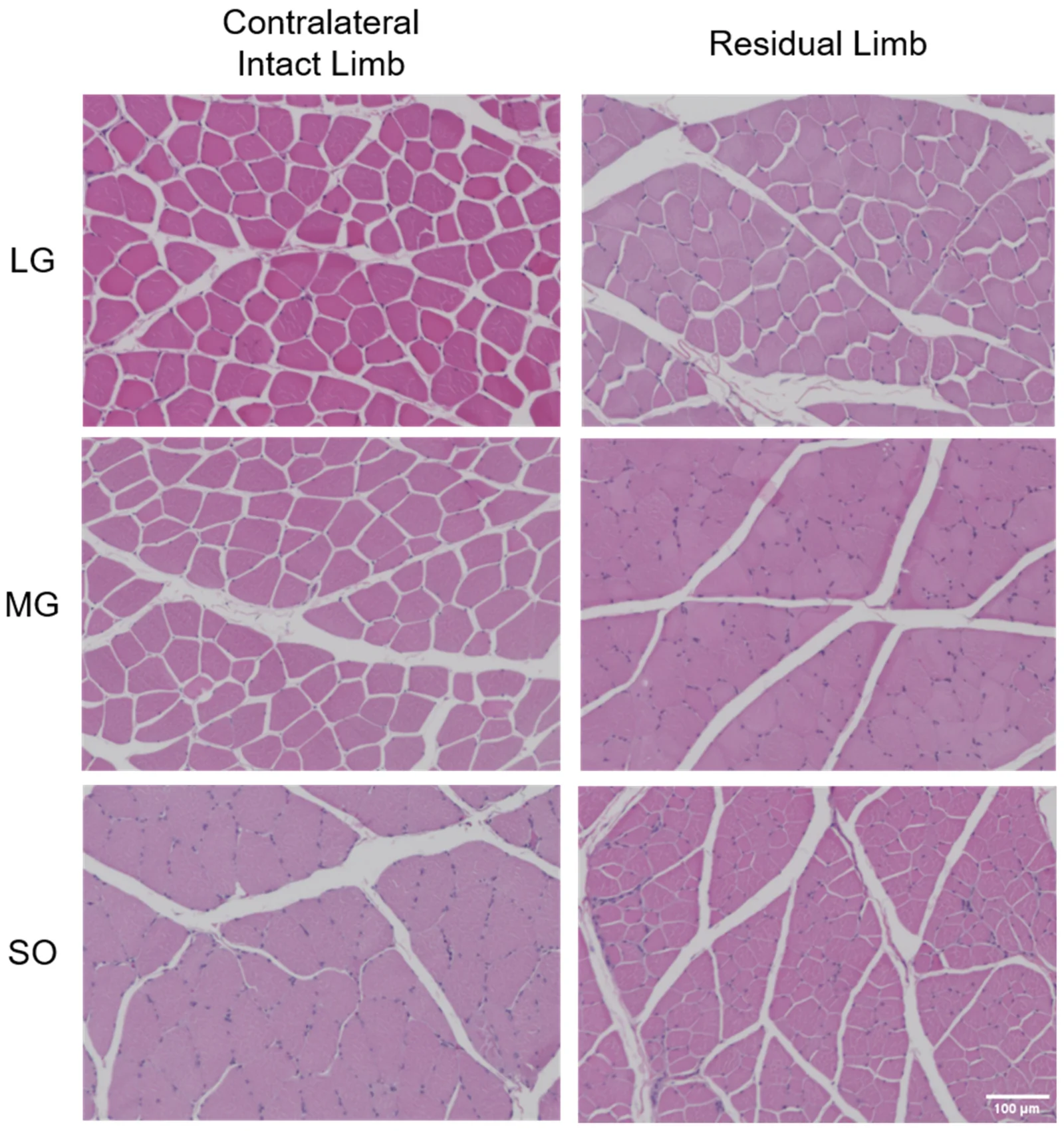
Muscle histology images of the lateral gastrocnemius (LG), medial gastrocnemius (MG), and soleus (SO) muscles from the contralateral intact and residual limbs of one rabbit. Muscles were sectioned perpendicular to the long axis of the fibers.

**Figure 3:**
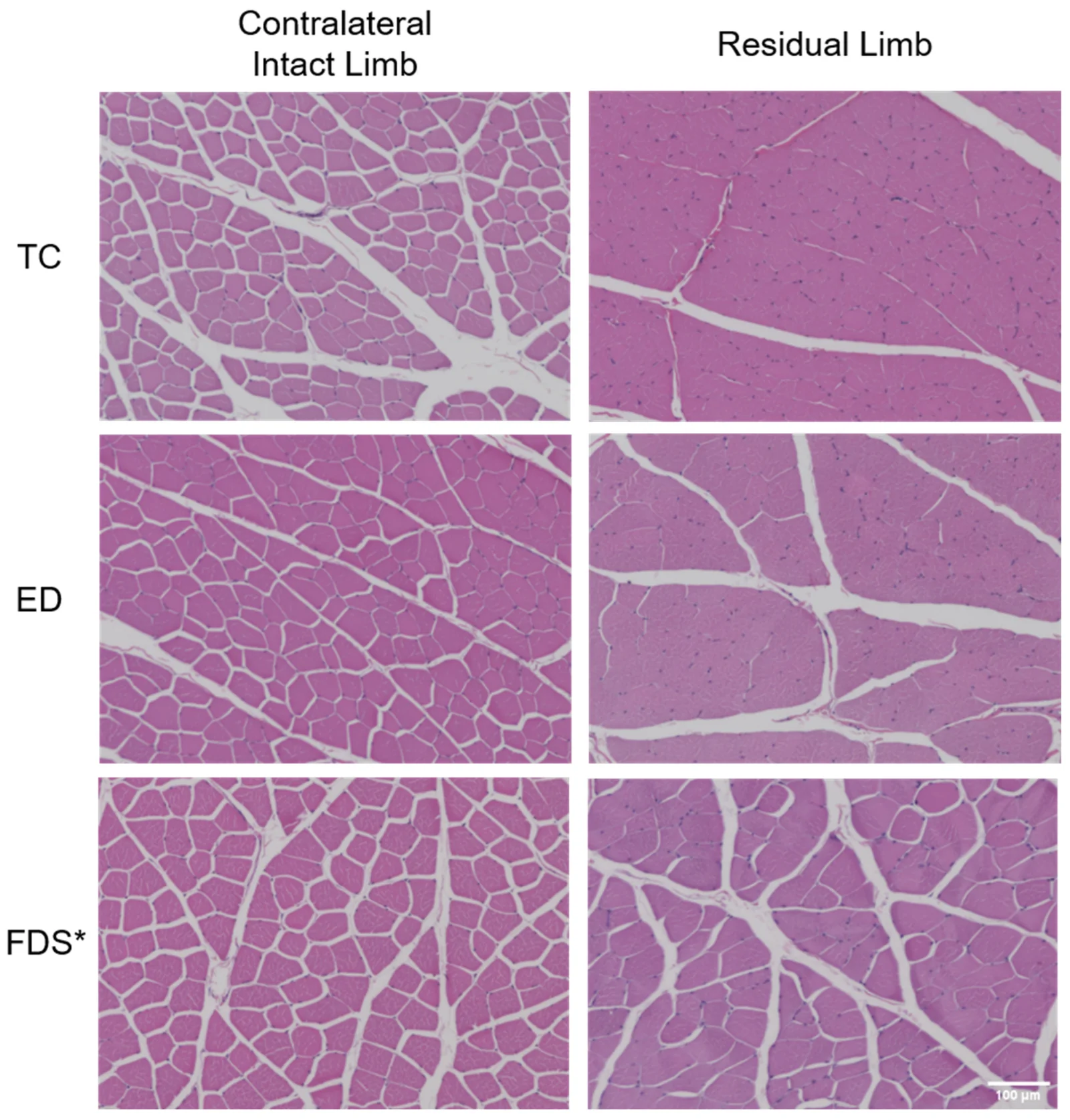
Muscle histology images of the tibialis cranialis (TC), extensor digitorum (ED), and flexor digitorum superficialis (FDS) muscles from the contralateral intact and residual limbs of one rabbit. Muscles were sectioned perpendicular to the long axis of the fibers. * Due to an error in muscle sectioning, the FDS muscle sections are from a different rabbit than the other muscle sections in Figure 2 and Figure 3.

Qualitatively, from the histology images of the MG and SO muscles (Figure 2), the fiber cross-sections appeared smaller and of more variable size in the residual limb than in the intact contralateral limb. Conversely, for the LG, ED, and FDS muscles (Figure 2 and Figure 3), fiber cross-sectional area size and size variability appeared similar between sides. Fiber cross-section shapes were qualitatively similar between sides for all muscles. Boundaries between adjacent fibers were difficult to visualize for the residual TC and ED muscles (Figure 3).

## 4. Discussion

Following amputation, residual muscles experience a combination of mechanical unloading, chronically altered (usually shortened) muscle length, and reduced muscle excursion. Skeletal muscles may experience similar conditions following tenotomy and joint immobilization, for which muscle architecture changes have been well characterized in animal models [13,17,18,19]. A common and expected muscle change with chronic unloading is atrophy, defined as a loss of muscle mass. All measured muscles in our study crossed the amputated ankle and, therefore, would be expected to have reduced load, excursion, and (for non-tethered muscles) in situ length. Correspondingly, all measured muscles had lower average mass, muscle length, and optimal fiber length in the residual limb compared to the contralateral intact limb, though differences were only significant for a few muscles. Previous studies in humans similarly reported that muscles that once crossed the missing joints have lower muscle volumes than in a healthy, intact limb [8,20]. One clinical factor affected by a loss of muscle mass and volume is residual limb volume and prosthesis socket fit. Changes in residual limb volume and shape are known problems that can lead to poor socket fit, gait instability, poor sensor contact (for myoelectric prostheses), and a risk of skin breakdown [3,20,21]. Therefore, there is a need for scalable interventions to prevent loss of muscle mass and volume to improve prosthesis socket fit, function, comfort, and health [22].

Both the LG and SO muscles had substantially lower (average percent difference > 30%) mass and optimal fiber length on the residual side compared to the contralateral intact side, though differences were only statistically significant for LG. Because the LG crosses the residual knee whereas the SO does not, we initially expected that residual-knee motion and hindlimb loading would protect the LG from degenerative changes. As little as 30 min of daily passive muscle excursion has been shown to prevent sarcomere loss in series [23]. Anecdotally, the rabbits used the residual limb during ambulation and weight bearing, although future studies should quantify hindlimb biomechanics to better characterize the relationship between limb use and muscle adaptation. Thus, while more direct, quantitative evidence is needed, crossing the residual knee did not appear to confer substantial protection against degenerative muscle changes in our amputation model.

Muscle fiber type is also thought to potentially influence a muscle’s susceptibility to degeneration during chronic reductions in loading and excursion. The soleus is composed primarily of type I, slow oxidative fibers [19], unlike the gastrocnemius [24]. Previous rabbit tenotomy studies have reported rapid and substantial degeneration of the soleus relative to other muscles as early as 2 weeks after injury [19]. Similarly, hindlimb-unloading studies have shown that the soleus undergoes greater reductions in muscle mass, fiber cross-sectional area, and oxidative capacity than muscles with a higher proportion of fast-twitch fibers [25,26]. Nevertheless, the pronounced adaptations observed in both the SO and LG suggest that fiber-type composition may explain less of the intermuscular variability in adaptation than we initially anticipated.

PCSA is directly proportional to the maximum isometric force a muscle can produce [17]. Despite lower muscle mass in all residual muscles, including two that were significantly lower, PCSA was generally preserved in all muscles except FDS. This is because PCSA is a function of muscle mass, pennation angle, and optimal fiber length (Equation (3)); for a given muscle mass, PCSA increases as either optimal fiber length or pennation angle decreases. On average, all residual muscles had lower optimal fiber length than their contralateral intact counterparts, and some also had lower pennation angles. Together, these architectural changes seemed to have offset the reduction in muscle mass, yielding a net increase in computed PCSA. Previous studies of immobilized hindlimb muscles have reported similar paradoxical increases in PCSA, attributable to disproportionately greater reductions in fiber length than in muscle mass [13,18]. However, despite PCSA being preserved, muscles with a lower optimal fiber length would produce active force over a narrower range of lengths, limiting their functional utility in the residual limb.

Although quantitative histological analysis of fiber cross-section morphology was beyond the scope of this pilot study, it may reveal cellular-level changes associated with alterations in muscle architecture and should therefore be prioritized in future studies. For example, other previous studies observed a coincidence of muscle atrophy and lower fiber cross-sectional area with conditions of functional impairment such as aging [27], joint immobilization [22], and limb unloading [26]. The difficulty in visualizing inter-fiber boundaries in the residual TC and ED muscles (Figure 3) may reflect early increases in extracellular connective tissue or interstitial edema, which have been reported in muscles subjected to disuse and unloading [28]; future studies using Masson’s trichrome or Sirius red staining could confirm whether fibrotic remodeling could explain the differences in boundary visibility. Beyond H&E staining, laminin/dystrophin immunofluorescence, fiber-type stains, NADH-TR or SDH, and atrophy- or NMJ-related immunostains could help further characterize unloading- and trauma-associated skeletal muscle remodeling after amputation [29,30,31,32,33]. Formalin-fixed, paraffin-embedded sections were used, which are compatible with H&E; however, fresh or fresh-frozen tissue would be needed to conduct immunofluorescence-based fiber-type or membrane-integrity staining.

Other qualitative signs of muscle degeneration beyond cross-sectional morphology were not apparent from the histology sections. Common histological indicators of muscle degeneration in H&E-stained sections include reduced eosin affinity (indicating lower protein content), centralized or crowded nuclei, and adipocyte infiltration within the epimysium or perifascicular space [34]. Notably, these signs are more characteristic of chronic or myopathic conditions than of early disuse or unloading, which may explain their absence at four weeks post-amputation. Future studies should quantify these indicators to more rigorously characterize and compare residual muscle tissue quality between sides.

Muscle changes following amputation are likely influenced by several factors, such as the level (i.e., location on the limb), cause, and nature (e.g., clean cut or blunt trauma) of amputation, surgical manipulations during revision amputation, and post-operative limb bandaging, movement, and loading, that may vary among patients. Some, such as surgical manipulations and post-operative conditions, can be controlled to some extent. To inform clinical practice and optimize outcomes, future studies should investigate the effect of controllable factors on muscle architecture and other relevant variables.

We expected that muscles attached to the distal end of the bone (TC, LG, MG, SO) would have more similar length between sides compared to unattached muscles (ED, FDS). This expectation was based on previous immobilization studies, which showed that muscles fixed at longer lengths experience less degeneration [13]. However, contrary to our expectation, mean optimal fiber length of the LG, MG, and SO was 44%, 32%, and 51% lower, respectively, on the residual side than on the intact contralateral side, with the raw bilateral difference for LG being statistically significant (*p* = 0.026). In situ muscle lengths were also, on average, lower in the residual limb across all muscles, suggesting that the attached muscles may have been at a chronically shortened length despite our attempt to preserve their length. These results should be interpreted cautiously, since we did not compare the same muscles between sutured and non-sutured conditions, and different muscles may respond differently. It is also possible that the sutured muscles were chronically shortened compared to pre-amputation, and future studies should verify this through intraoperative measurement of muscle lengths. Surgeons often manipulate the residual muscles in various ways to shape the residual limb for prosthesis sockets [35]. Thus, surgical muscle attachment may be a simple and practical means to preserve muscle architecture if it proves effective.

Residual muscle degeneration such as that preliminarily indicated by our pilot, exploratory study could have significant functional implications for people with amputation beyond muscle volume changes and poor socket fit. For example, muscle degeneration degrades voluntary muscle activation [36], which could impair amputees’ function with myoelectric prostheses. Since even residual muscles that crossed the knee (e.g., lateral gastrocnemius) were degenerated, residual joint function could be impaired. For prostheses that are physically attached to external prostheses (via cineplasty [5,6]) or internal endoprostheses [7], our study preliminarily suggests that the attached muscles would be able to generate active force over a narrower range of lengths; if this were the case, the patient would potentially have less range of motion and less strength at most joint postures with the residual muscle attached to the prosthesis than if the same muscle were attached in its normal, intact condition. Since the attached residual muscles would resume experiencing more force and length changes, it is likely that the muscles would remodel in such a way as to increase their force-generating capacity by regaining mass and optimal fiber length, an expectation supported by a previous study [5]. This is a potential advantage of muscle-driven prostheses and one that should be confirmed in future studies.

Our pilot study had several limitations and, thus, the results should be interpreted with caution:

1. Despite our study’s small sample size, we performed multiple statistical tests to identify potential trends in muscle property differences between sides and among muscles. Multiple statistical tests can increase the type I error (false positive) rate. Moreover, our normality test grouped data across animals, muscles, and parameters, while the statistical tests compared different muscles for each parameter separately. Though we adjusted *p*-values to reduce the risk of type I error, larger follow-up studies will be needed to confirm and extend these findings with adequate statistical power.
2. We used the contralateral intact limb as the experimental control; however, the rabbits’ increased reliance on the intact limb post-amputation may have caused confounding muscle changes (e.g., hypertrophy), and we did not obtain pre-amputation baseline measurements to confirm bilateral symmetry. Muscle architecture values from healthy control rabbits of similar size and age would serve as a better indicator of normal values, though such values have been reported in the literature [15].
3. Some variability among animals may have been attributable to measurement variation among samples or researchers, such as subtle differences in sample location; for example, pennation angle is known to vary considerably within a muscle [37], so great care was taken to measure at a consistent location across animals. To capture the within-muscle variability in architecture, future studies could make measurements at different locations within each muscle. Data variability may also have been induced by unmonitored inter-subject variation in surgical or post-surgical procedures, post-surgical mobility and limb loading, or other factors that could potentially affect muscle architecture; these factors should either be more rigorously controlled or monitored in future studies to reduce or account for such variations in the results.
4. We measured muscle architecture at only one post-amputation time point; future studies should include additional time points to better characterize the rate of architectural change and the point at which changes plateau. We measured muscle architecture as an indirect indicator of force-generating capacity, but did not directly measure muscle forces or contractile properties; future studies should complement architectural measurements with in situ or in vitro force testing to confirm that the changes reported here translate to the expected functional deficits.
5. The operated limb and order of muscle measurements were not randomized. The researchers who performed the muscle measurements were not blind to the muscle type or side. The intra-rater and inter-rater reliability of the measurements was not assessed. These limitations could have introduced bias or other errors in our measurements.
6. Our histological analysis was qualitative and descriptive. Quantitative measures of fiber cross-sectional area, fiber type proportions, and intramuscular adipose content would provide a more complete and rigorous characterization of residual muscle tissue quality.
7. Muscles were fixed in formalin prior to dissection, which may have introduced dimensional changes that affected the absolute values of measured architectural parameters; however, since both residual and intact limbs received the same fixation, it should not substantially bias the within-subject differences reported here.

In conclusion, our preliminary data suggest that ankle disarticulation may produce measurable architectural degeneration in residual hindlimb muscles within four weeks, characterized by substantial atrophy and reduced optimal fiber length. Qualitatively, from histology images, the MG and SO muscles appeared to have smaller muscle fiber cross-sections in the residual limb. Degeneration was most severe in the SO and LG muscles, despite differences in musculoskeletal geometry and fiber type composition between these muscles. Even muscles whose tendons were sutured to bone had relatively less muscle length and optimal fiber length on the residual side, suggesting that tendon anchoring alone is insufficient to preserve fiber-level architecture. Despite lower mass, several muscles showed preserved or paradoxically increased PCSA, possibly attributable to disproportionately greater reductions in optimal fiber length than in mass. Though more rigorous follow-up studies are needed to confirm our findings, they have potential direct implications for residual limb and prosthetic function, such as poorer quality of myoelectric signals for prosthesis control and less functional range of motion and strength for muscle-attached prostheses. We expect that the preliminary measurements from this pilot, exploratory study will inform the design of a larger, more rigorous follow-up study, including power analyses to estimate the required sample size. Our findings motivate additional research to (1) characterize the rate and extent of post-amputation muscle degeneration across amputation levels and timepoints, (2) identify the mechanical and biological factors that determine a muscle’s susceptibility to degeneration, and (3) evaluate practical surgical and rehabilitative strategies to preserve residual muscle architecture and thereby improve prosthetic and residual limb function in people with amputation.

## Figures and Tables

**Table 1:** Muscle architecture summary statistics, including mean and standard deviation of raw values from the intact and residual limbs, raw difference (Equation (4)), and percent difference (Equation (5)); FDR-adjusted *p*-values from statistical 1-sample *t*-tests of raw difference; and 95% confidence intervals of the raw difference.

Variable	Muscle	Intact Limb		Residual Limb		Raw Difference (Residual − Intact)		Percent Difference		FDR-Adjusted *p*-Value *	95% Confidence Interval of Raw Difference
		Mean	Std Dev	Mean	Std Dev	Mean	Std Dev	Mean	Std Dev		
Mass (grams)	LG	5.51	0.92	3.53	1.15	−1.97	0.67	−37	14	0.029	(−2.80, −1.14)
	MG	5.70	1.24	3.98	0.89	−1.73	1.41	−28	20	0.129	(−3.48, 0.02)
	SO	2.65	0.57	0.92	0.51	−1.73	0.93	−63	26	0.072	(−2.88, −0.59)
	TC	3.08	0.71	2.83	0.50	−0.25	0.30	−7	9	0.250	(−0.62, 0.13)
	ED	3.08	0.71	2.34	0.25	−0.74	0.51	−21	16	0.112	(−1.38, −0.10)
	FDS	4.35	0.90	2.46	0.41	−1.89	0.54	−43	5	0.026	(−2.57, −1.22)
Length (mm)	LG	60.15	3.42	52.42	11.33	−7.74	13.27	−12	23	0.391	(−24.22, 8.74)
	MG	65.37	6.22	57.27	7.51	−8.10	3.55	−13	6	0.049	(−12.51, −3.69)
	SO	62.62	10.20	54.54	12.33	−8.08	5.09	−13	10	0.111	(−14.4, −1.76)
	TC	65.42	7.15	57.30	5.53	−8.12	5.29	−12	7	0.112	(−14.69, −1.55)
	ED	72.92	8.95	70.68	5.21	−2.24	7.48	−2	11	0.631	(−11.53, 7.06)
	FDS	73.00	9.26	60.22	4.70	−12.78	11.64	−16	17	0.155	(−27.24, 1.67)
Pennation Angle (degrees)	LG	26.19	4.49	36.08	9.28	9.89	7.78	38	31	0.129	(0.22, 19.56)
	MG	28.51	2.90	28.29	7.49	−0.22	9.95	2	34	0.963	(−12.58, 12.13)
	SO	28.70	11.48	26.70	5.00	−2.01	11.38	5	38	0.768	(−16.14, 12.13)
	TC	7.81	7.74	4.87	0.91	−2.93	7.86	9	71	0.574	(−12.70, 6.83)
	ED	19.43	5.54	24.06	7.08	4.62	11.72	47	104	0.574	(−9.93, 19.18)
	FDS	29.28	3.99	22.21	2.79	−7.06	2.60	−24	7	0.031	(−10.29, −3.83)
Fiber Length (mm)	LG	12.23	1.94	7.87	0.99	−4.36	1.33	−35	6	0.026	(−6.00, −2.71)
	MG	12.66	1.52	9.29	2.20	−3.37	2.85	−26	23	0.133	(−6.91, 0.17)
	SO	10.97	2.45	6.32	2.95	−4.65	4.85	−38	37	0.180	(−10.66, 1.37)
	TC	43.82	15.91	32.70	12.44	−11.12	18.56	−12	57	0.391	(−34.16, 11.93)
	ED	15.55	2.81	10.16	0.52	−5.39	2.81	−33	15	0.072	(−8.88, −1.90)
	FDS	9.32	1.36	9.74	1.51	0.42	0.66	5	7	0.369	(−0.40, 1.25)
Sarcomere Length (μm)	LG	1.80	0.19	2.12	0.30	0.32	0.30	18	17	0.159	(−0.06, 0.69)
	MG	1.83	0.24	2.04	0.26	0.21	0.20	12	11	0.159	(−0.04, 0.46)
	SO	1.74	0.31	2.42	0.38	0.68	0.66	45	50	0.159	(−0.14, 1.49)
	TC	2.25	0.18	2.19	0.23	−0.06	0.28	−2	12	0.705	(−0.41, 0.28)
	ED	2.58	0.32	2.25	0.47	−0.33	0.47	−12	16	0.320	(−0.91, 0.25)
	FDS	1.72	0.28	1.93	0.12	0.21	0.37	16	27	0.391	(−0.25, 0.67)
Physiologic Cross-Sectional Area (cm^2^)	LG	3.18	0.73	3.24	1.01	0.06	0.76	3	24	0.895	(−0.89, 1.01)
	MG	3.07	0.40	3.32	0.70	0.25	0.95	11	35	0.668	(−0.93, 1.42)
	SO	1.68	0.87	1.33	0.24	−0.35	1.09	−4	42	0.628	(−1.71, 1.00)
	TC	0.73	0.22	1.05	0.88	0.33	0.86	45	104	0.574	(−0.74, 1.39)
	ED	2.05	0.29	2.02	0.48	−0.03	0.32	−2	15	0.895	(−0.42, 0.37)
	FDS	3.06	0.99	1.98	0.42	−1.08	0.85	−33	15	0.129	(−2.14, −0.02)
Optimal Fiber Length (mm)	LG	14.96	2.03	8.23	0.82	−6.74	2.05	−44	8	0.026	(−9.28, −4.19)
	MG	15.61	3.56	10.17	2.61	−5.44	4.18	−32	28	0.129	(−10.64, −0.25)
	SO	14.22	3.88	5.93	3.19	−8.30	6.75	−51	40	0.129	(−16.68, 0.09)
	TC	43.28	16.01	33.82	14.53	−9.46	17.25	−9	55	0.403	(−30.88, 11.96)
	ED	13.42	2.76	10.28	2.15	−3.14	2.12	−22	15	0.112	(−5.77, −0.51)
	FDS	12.03	1.36	11.21	2.23	−0.82	2.75	−6	23	0.631	(−4.24, 2.60)

* based on 1-sample *t*-test of raw difference with false discovery rate (FDR) to correct for multiple tests.

## Data Availability

The original contributions presented in this study are included in the article/Supplementary Materials. Further inquiries can be directed to the corresponding author.

## Supplementary Materials

The following supporting information can be downloaded at: https://www.mdpi.com/article/10.3390/muscles5030065/s1, “muscle data raw differences.txt” is a tab-delimited data file containing the raw bilateral difference values for each muscle, animal, and muscle architecture parameter. “README.txt” is a text file that lists the filename, file format, and muscle ID in the data file.
